# Transition to Adulthood Autonomy Scale for Young People: Design and Validation

**DOI:** 10.3389/fpsyg.2020.00457

**Published:** 2020-03-20

**Authors:** Teresita Bernal Romero, Miguel Melendro, Claudia Charry

**Affiliations:** ^1^Faculty of Psychology, Universidad Santo Tomás, Bogotá, Colombia; ^2^Faculty of Education, UNED, Madrid, Spain; ^3^Faculty of Psychology, Universidad Santo Tomás, Bogotá, Colombia

**Keywords:** autonomy, young people, assessment, orientation, transition to adulthood, validation, scale

## Abstract

Given that autonomy is a fundamental process in the transition to adulthood, there are several scales that measure the concept as a main construct or a constituent feature of broader constructs. However, most of these scales are based on a notion of autonomy focused on the individual, while the proposed scale aims to incorporate the idea of the individual mediated by others and society. This article aims to show the results of the design and validation process of the Transition to Adulthood Autonomy Scale (EDATVA), which was developed using this approach. A group of 61 items with a Likert-type response scale of four options was used on a sample of 1,148 Spanish and Colombian individuals, aged between 16 and 21. A systematic process was performed using an exploratory factorial analysis. Additional indexes were calculated from the Rasch Model. The matrices obtained from the factorial analysis gave rise to a 4-factor structure comprising a total of 19 items with weights >0.3. In the case of Spain, the KMO test returned a value of 0.80 and in the case of Colombia, 0.83. In the Rasch model, the Item Separation Reliability (0.99) indicates that the items constitute a well-defined variable that meets the local independence assumption. Cronbach's alpha for the Spanish sample was 0.86 and for the Colombian sample 0.85. In conclusion, this new scale consists of four dimensions: self-organization, understanding context, critical thinking, and socio-political engagement. The scale is easy to use and interpret, especially considering the age range of the target population and its possible uses within the contexts of assessing and intervening in young people's behavior. Due to its characteristics, it can be used in family, educational, and social contexts. This scale is valuable for research because its optimal psychometric properties provide an alternative way of understanding autonomy.

## Introduction

The transition to adulthood is an emerging and rapidly growing field of research, which aims to address the many challenges young people face during their development (Casal et al., 2015; Melendro and Rodríguez-Bravo, 2015; Courtney et al., 2017; Mann-Feder and Goyette, 2019). In order to achieve a successful transition from adolescence to adulthood, young people must endeavor to master the processes linked to autonomy. These processes enable young people to make decisions in complex situations that will have a fundamental bearing on their adult lives. Such situations include moving away from the family environment to build an independent life, potentially raising a new family, entering the labor market, continuing in education, organizing personal finances, self-care, forming social networks, solving everyday problems, inter alia (Álvarez, 2015; Esteinou, 2015; Ferraris and Martínez, 2015; Bernal, 2016; Moleiro et al., 2016; Garberoglio et al., 2017; Hernández, 2017; Linne, 2018; Okpych and Courtney, 2019).

Given the widely accepted importance of the processes in the human lifecycle linked to autonomy, a variety of measures have been designed to assess their impact. Below is a review of just some of the measures that have been used as references to design the model used in this study, the Transition to Adulthood Autonomy Scale, in Spanish *Escala de Autonom*í*a en el Tránsito a la Vida Adulta* or EDATVA.

To begin with, there are a series of questionnaires that assess autonomy based on adolescents' self- perception. This is the case of the Adolescent Autonomy Questionnaire (Noom, 1999), which aims to assess functional, emotional, and attitudinal autonomy from the perspective of adolescents aged 12–15. The Adolescent Autonomy Behavior Questionnaire (Fleming, 2005), used with individuals aged 12–17, also focuses on young people's self-assessment of their own actions. A more recent measure, but still in the revision process, is the Autonomy Questionnaire (Martínez-Torres and Ojeda-Gutiérrez, 2016), which aims to measure autonomy in reference to four contexts: education center; personal milieu; academic learning, and social environment. Lastly, the Ansell-Casey Life Skills Assessment (ACLSA) scale (Nollan et al., 2001) aims to assess the functional autonomy of young people aged 14–21; the questionnaire must be completed by the individual and their guardian, and includes the following dimensions: daily living activities, self-care and healthcare, housing and community resources, budgeting and paying bills, maintaining healthy relationships, and work and study habits.

Other types of measures assess emotional autonomy. One of the most important is the Emotional Autonomy Scale (EAS) (Steinberg and Silverberg, 1986), which measures four dimensions in adolescents aged 14–18: individuation, deidealization of parents, non-dependence on parents, and perceptions of parents as individuals. In turn, other measures focus on cognitive autonomy, such as the Cognitive Autonomy and Self-Evaluation (CASE) inventory (Beckert, 2007), which is targeted at adolescents and has includes the following components: evaluative thinking, voicing opinions, decision making and comparative validation.

There are also a number of models that deal with measuring autonomy in interrelation to other constructs, such as the Sociotropy-Autonomy Scale (SAS), which comprises two subscales: sociotropy and autonomy. The first measures sociopathic attitudes, such as fear of criticism and rejection, and a preference for affiliation. The second measures autonomic attitudes through individualistic achievement and freedom from control (Beck et al., 1983). Also in this group is the Autonomy-Connectedness Scale (ACS-30) (Bekker and van Assen, 2006), which comprises three sub-scales for individuals aged 18–59: self-awareness, sensitivity to others, and capacity for managing new situations. And lastly, the Satisfaction of Proximity/Autonomy Needs Scale (ERSN-P) (Bernardo and Branco, 2015), targeted at young adults, measures the satisfaction of needs from two subscales: proximity and autonomy.

Another group of models measures autonomy as part of a larger construct. É*preuve de Développement Psychosocial*, DPS-66 (Cited in Behar and Forns, 1984) measures three dimensions: behavioral autonomy, social integration and social intelligence, although it focuses more on childhood than adolescence. These dimensions, as discussed in Behar and Forns (1984), were later included in É*preuve de Développement Psychosocial* (EDPS/74) (Coulbaut, 1981), which measures psychosocial maturity in adolescents aged 12–18. In turn, the Psychological Maturity Questionnaire (Morales et al., 2012) measures the capacity of young people aged 15–18 to make responsible decisions, in other words, to analyze the consequences before making a decision. This model comprises three dimensions: orientation toward employment, autonomy, and identity. Also important to mention is the Model of Psychological Well-being (Ryff, 1989). Although this model focuses on measuring psychological well-being, it presents a subscale that measures autonomy in the form of independence, understood as resisting social pressure in order to make decisions based on personal standards. The Motivational Profile Analysis (APM) (Valderrama et al., 2015) can be used on individuals from the age of 16 and measures 10 dimensions: affiliation, autonomy, power, cooperation, achievement, hedonism, exploration, security, contribution, and conservation.

And lastly, there are also a series of models that measure constructs that can be considered similar to autonomy. The Emotional Dependency Questionnaire (Lemos and Londoño, 2006) measures young people's (aged 16–18) level of dependence based on self-perception, perception of others, threats, and interpersonal strategies. The Sociopersonal Factors Test for Labor Insertion (Martínez-Rodríguez and Carmona, 2010), also designed for young people, is structured in five sections. One of which, personal qualities to access employment, recognizes autonomy as an important variable, although it should be noted that in this case autonomy is a feature that is exclusively related to employment. The Adaptive Behavior Assessment System (ABAS II) (Oakland and Harrison, 2011) does not measure autonomy, but gauges adaptive behavior from birth to age 89 with the aim of assessing an individual's abilities to live independently. In this model, adaptive behavior is measured based on functional skills such as: communication, use of community resources, functional academic skills, home and study life, health and safety, leisure, self-care, self-direction, and employment.

After reviewing these and other methods of measuring autonomy, we identified three different approaches, which coincide with those described by various authors as subjective spaces or representations of reality (Boudon, 1995; Krakov and Pachuk, 1998; Jodelet, 2009; Kaës, 2015; Ferreira et al., 2016). This, in turn, infers different approaches to autonomy: (1) as a personal, subjective or intrasubjective process; (2) as an intersubjective construction, which requires relationships with others to make sense, and (3) from a trans-subjective perspective, as the interaction between individuals and the society in which they live.

In the first, the personal or intrasubjective approach, the definitions of autonomy focus on the individual's capacity and skills to self-govern (Sandhu and Kaur, 2012; Conill, 2013; Bussières et al., 2015; Majorano et al., 2015; Fernández-García, 2016), which entails processes of self-determination, self-sufficiency and self-regulation (Markus and Wurf, 1987). Authors such as Inguglia et al. (2015) define this same approach from the perspective of an individual's will and self-organization. The intrasubjective approach has been primarily used in designing measures of autonomy, some of the most important being: the Adolescent Autonomy Questionnaire (Noom, 1999), a model that integrates the principal intrasubjective perspectives (attitudinal, emotional and functional); the Sociotropy-Autonomy Scale (SAS) (Beck et al., 1983), which measures sociotropy and autonomy, and the Model of Psychological Well-being (Ryff, 1989), a subscale which measures autonomy in the form of independence, understood as resisting social pressure in order to make decisions based on personal standards (García-Alandete, 2013).

The second, the relational or intersubjective approach, analyzes different aspects of relationships between individuals such as: cooperating in the development of personal attitudes, knowledge and competences (Thomas and Da Costa, 2014); promoting the transmission and dissemination of information and mechanisms of reciprocal influence (Jodelet, 2009), and encouraging “co-autonomy,” which involves compassion and reciprocal recognition (Cortina, 2017) especially in the family (Esteinou, 2015; Van Petegem et al., 2015; Duineveld et al., 2017; Garberoglio et al., 2017; Kiang and Bhattacharjee, 2018) and educational environments (Barker, 2014; Bustamante et al., 2014; Hein and Jõesaar, 2015; Antunes and Correia, 2016) both of which are privileged spaces for the study of this type of autonomy. This approach, which appears on many occasions to complement the intrasubjective approach or other constructs relating to autonomy, has given rise to models which provide interesting and differential contributions, such as: the Autonomy-Connectedness Scale (ACS-30) (Bekker and van Assen, 2006) which includes a subscale focusing on sensitivity to others, and the College-Student Scale/Parental Behavior Questionnaire/Perceptions of Parents Scale (Campione-Barr et al., 2015), which focus on family relationships between parents and children.

The third, the social or trans-subjective approach, studies the processes of autonomy from the perspective of the interaction between individuals and their society, as well as the important influence of educational, social, media, economic, and political factors as facilitators or obstacles of decision making (Cáliz et al., 2013). In this approach, the definitions of autonomy focus fundamentally on the interaction of the individual with other contexts in two complementary ways:

(1) the construction of autonomy as a product of the individual's interactions with the environment, including the idea of social responsibility (Mazo, 2012; D'Angelo, 2013; Posada, 2013) or as a factor of social transformation (Muñoz-López and Alvarado, 2011), and (2) the limits placed on autonomy that are generated by the different contexts in which the individual interacts (Cáliz et al., 2013; Álvarez, 2015). There are few models that follow this focus in their structure and item design, and limited significant contributions. Notwithstanding, worth highlighting are the Ansell-Casey Life Skills Assessments (ACLSA) (Nollan et al., 2001), which assesses the individual's relationship with community resources, and *Une Épreuve de Développement Psychosocial* (EDPS/74) (Coulbaut, 1981), which is based on three dimensions, one of which is “social integration,” “which analyses the individual's participation in collective life” (Behar and Forns, 1984).

Consistent with the literature and measures reviewed and mentioned above, the objective of this study was to determine the design and validation process of a new scale, the Transition to Adulthood Autonomy Scale (EDATVA). This scale brings to the construct of autonomy the idea that an individual's decisions are also mediated by others and society, and not just by the individual themselves. Moreover, this process occurs at a fundamental evolutionary, social and educational period in the lives of adolescents and young people—the transition to adulthood.

## Study 1: Scale Design and Construction

### Materials and Methods

#### Participants

In the first study, in which an initial EDATVA design was used, 160 young people aged 17–19 from Colombia were selected using convenience sampling (Mean = 18.2, SD = 0.7), 106 female (66.3%), 53 male (33.1%), and 1 transgender (0.6%). Of this group, 85% were studying, 12.4% working and studying, 1.3% working, and 1.3% were not engaged in any of the two activities. The majority were living with their parents or relatives (92%) and 5% stated that they were living alone. The only selection criteria stipulated for EDATVA was age range.

#### EDATVA Item Proposal

The initial proposal for the EDATVA items was obtained by following the steps below.

##### Concept Definition

A literature review was performed to determine how different authors have defined autonomy. For this study, autonomy is understood as a complex process, which is in continuous development throughout an individual's lifetime and via interaction with others (Muñoz-López and Alvarado, 2011; Cáliz et al., 2013; Posada, 2013) and involves procedural elements such as: (1) to question and reflect on one's own life in relation to others, (2) to make interdependent decisions and assume the consequences, and (3) to practice self-eco-organization (relating to oneself, others and society).

Based on these characteristics, we created three dimensions for autonomy. The first considers the processes of reflection and decision making with respect to the subject. The second considers these processes as regards close “others” (family, friends, partner), and the third as regards community and social references.

##### Review of Measures and Item Selection

A review of existing measures aimed at assessing autonomy was performed. On the basis of this review, a scale was drawn up to group the previously mentioned conceptual elements. In principal, the elements, which were deemed fundamental to the study, were targeted at young people in their transition to adulthood.

A group of seven experts and researchers, with extensive experience in working with young people and academic backgrounds in the subject of autonomy and the transition to adulthood, designed a total of 68 items taking into account the conceptual, procedural, and self-eco-organization elements in the proposal. They also took into consideration items and dimensions from other measures aimed at assessing autonomy or similar constructs (Nollan et al., 2001; Fleming, 2005; Díaz et al., 2006; Aparicio, 2010; Gobierno, 2012; Larimore, 2012; Berzin et al., 2014; Verdugo et al., 2014; Bussières et al., 2015; Oudekerk et al., 2015; García, 2016; Moleiro et al., 2016).

The aim was to generate a group of items three times larger than those required for the scale, following recommendations from several authors on the subject (Aiken and Groth-Marnat, 2005; Wendler and Walker, 2006).

##### Peer Review

For this stage, a group of experts was selected based on their knowledge and experience on the subject of autonomy and working with young people, either as researchers or as caregivers. Experts with experience in developing tests and psychometry were also included. In total, the panel consisted of seven experts, three Spanish and four Colombian. Each was given a protocol to assess the wording, understanding and coherence of the 68 items, scoring each on a scale from 1 to 5. In addition, they were able to record their observations and suggestions for the items on the format. Indices were calculated by unanimous agreement of the panel (Aiken's V) for each of the aspects assessed. All the scores obtained with Aiken's V were >0.8 (in wording and coherence >0.8, and in comprehension >0.9). In addition, small changes were made to the wording of some of the items and one was relocated to another dimension which the panel considered a better fit.

##### Pilot Questionnaire Design

In order to categorize the sample, some questions on sociodemographic data were included. A 4-point Likert scale was used for the EDATVA items: from 1 strongly disagree to 4 strongly agree. The items were organized in an online questionnaire to facilitate use, and a printed format was made available for those participants without access to an electronic device.

#### Procedure

A number of institutions were approached and negotiations held in order to process the corresponding guarantees required for young people to participate in the study. In addition, informed consents and approvals (depending on the circumstances) were given to each participant and/or their legal representative. Most of the submissions were administered in groups in sessions that lasted approximately 15 min. Printed and online forms were available, depending on the needs of the participants.

### Data Analysis

A number of indices were calculated to assess how the questions would initially perform, including the discrimination index, the test-item correlation and Cronbach's alpha. In addition, the comments made by some of the participants about the items on the survey were reviewed.

### Results and Discussion

The following criteria were taken into account to interpret the indices: (1) items whose discrimination index was <0.2 (Ebel and Frisbie, 1986); (2) items with test-item correlation values >0.30, consistent with Nunnally (1970), and (3) items that, when eliminated, give rise to improvements in Cronbach's alpha for the test at 0.80.

As a result, a total of seven items were excluded from the original 68 items: “I'm capable of living independently from my family”; “I decide how to spend my money”; “I'm capable of avoiding harmful substance consumption (tobacco, alcohol, drugs)”; “My mistakes help me to learn”; “The best groups are those where all the have the same opinion”; “I value what I post on social media,” and “I don't participate in associations or social organizations because they're not worth it.”

None of the participants expressed difficulty in understanding the items on the survey. However, they did make observations or comments about some of the sociodemographic questions.

Thus, the initial structure proposed for EDATVA contained a scale of 61 items, distributed in three dimensions: (1) autonomy centered on the subject (intrasubjective), comprising 21 items; (2) autonomy in relation to close “others” (intersubjective), comprising 23 items, and (3) autonomy in relation to community and society (trans-subjective), comprising 17 items.

The design aims to reach a balance between items relating to the intra, inter and trans-subjective approaches mentioned in the introduction to this paper. The incorporation of the latter, which is relatively infrequent in autonomy scales, constitutes one of EDATVA's contributions to the field.

This approach responds to the reconceptualization of the construct of autonomy, understood as a complex process based on the assumption that the subject makes decisions mediated by others and by society. It is a key approach to contextualize and interpret the results obtained in the period for which EDATVA is designed: the transition to adulthood between the ages of 16–21.

## Study 2: Validation

In order to verify the structure, composition and reliability of the 61-item EDATVA (construct validity), data were collected from a new, much larger sample and an exploratory factorial analysis was performed. Some indices were also calculated using the Rasch model.

### Materials and Methods

#### Participants

The sample consisted of 1,148 young people, 508 (44.3%) Spanish and 640 (55.7%) Colombian selected using convenience sampling, with ages ranging from 16 to 21 (M = 18.2; SD = 1.8). The total group comprised 690 females (60.1%), 456 males (39.7%) and two participants who did not complete the data. Less than half of the sample (41%) consisted of university students, and nearly 56% were in some level of secondary education. A total of 2.5% stated that they were working, 76.2% exclusively engaged with study, 18.1% working and studying, and 3.2% were not engaged with either of the two activities. Lastly, 83.3% of the participants stated that they were living with their parents or relatives, and 8.7% in out-of-home placements.

In addition to age, literacy was taken into account as a criterion for inclusion, as it was a prerequisite for completing the survey. In turn, the presence of physical or mental functional diversity was considered an exclusion criterion, given that this aspect could give rise to processes of autonomy with very different characteristics. In order to verify this criterion, data was provided by the participants' teachers or caregivers from the various institutions participating in the study.

In order to obtain the sample, contact was made with various types of educational institutions, state agencies responsible for the care of young people at risk, and selected companies and social entities. It also involved applying for the corresponding permits, if and when applicable.

#### Procedure and Measures

Institutions were again approached and discussions held in order to contact potential participants. Informed consent from each of the participants was obtained and they were presented with the survey, which began with a section of sociodemographic questions that were followed by the EDATVA items. The survey was presented in its modified version following the results of Study 1: 61-items distributed in three dimensions: (1) autonomy focused on the subject, (2) autonomy in relation to close “others,” and (3) autonomy in relation to community and society. On this occasion, most surveys were completed using the printed form; the online version was only used in those cases where there was easy access to a computer or electronic device. Participants took an average of 15 min to complete the entire survey.

### Data Analysis

An exploratory factorial analysis (EFA) was performed for the total sample and for each country. Despite having a large number of items (61), the sample size (both in the total sample and at country level) was within the limits recommended for this method (Lloret-Segura et al., 2014). Cronbach's alpha and test-item correlations were calculated. Several indices were also calculated using the Rasch model owing to how they might contribute to the item analysis (Bechger et al., 2003; Abedalaziz and Leng, 2013) once the corresponding fit for the model was verified according to Linacre (2019)—infit and outfit values between 0.5 and 1.5 (see Table 1).

**Table 1:** Rasch fit statistics for selected items.

**Item**	**Spanish sample**		**Colombian sample**	
	**Infit**	**Outfit**	**Infit**	**Outfit**
25	0.74	0.72	0.78	0.75
26	0.75	0.71	0.85	0.79
27	0.74	0.74	0.68	0.69
28	0.70	0.70	0.57	0.57
29	0.64	0.65	0.68	0.67
35	0.91	0.91	0.85	0.80
56	0.82	0.80	0.69	0.70
57	0.85	0.82	0.76	0.75
49	0.73	0.68	0.68	0.67
59	1.19	1.11	1.07	1.11
50	0.84	0.84	0.77	0.76
51	0.97	0.99	0.93	0.91
52	0.92	0.93	0.83	0.83
36	1.07	1.11	1.06	1.06
40	1.09	1.08	0.92	0.90
53	1.15	1.18	1.00	1.00
54	1.36	1.41	1.19	1.21
55	1.04	1.04	1.01	1.01
9	0.79	0.79	0.87	0.88

### Results and Discussion

Test-item correlations were calculated to identify values <0.30, consistent with Nunnally (1970). Only three items with values below this limit were maintained (items 8, 14, and 15) when analyzing the groups separately (Colombia and Spain). Furthermore, in the total sample the three items contributed to good indices and their content was considered significant for the scale. The items would later show their significance in the constitution of the definitive factors in the exploratory factorial analysis (EFA).

The correlations between the items and measures obtained from the Rasch model were also analyzed. The criterion for positive correlations was considered >0.2 (Linacre, 2019). As the Rasch model assumes that all items are aligned in the same direction in the latent variable, it is vital that all items meet this criterion.

#### Exploratory Factorial Analysis

After refining the initial results, the matrices obtained gave rise to four consistent factors and their corresponding items (19 in total) with weights >0.3 in all cases (see Table 2). In the case of Spain, the KMO index returned a value of 0.800, where the first four factors explain 30.098%. In the case of Colombia, the KMO index returned a value of 0.834, where the first four factors explain 28.783%. Other groupings were also observed in neighboring components. However, they did not contribute significantly to the increase in the explained variance and included items which had specific issues. The fifth component in Colombia's matrix (equivalent to the eighth component in Spain's matrix) contained four items. On reviewing the statements, it was observed that they had a high degree of social desirability bias (e.g., “the country's problems should be solved by the government alone”). As a result, it was decided not to include them in the final selection. There was also a group of items in the sixth component of Colombia's matrix (equivalent to the sixth component in Spain's matrix) that alluded to independence in romantic relationships. However, this is related to the second component that was selected when defining the concept of autonomy (see Study 1), in other words, it is grouped with the processes of decision and reflection in relation to “others.” This dimension includes not only romantic partners, but also family and friends. Consequently, the component became too specific with respect to the objective of the study. And lastly, the seventh component did not contribute significantly to the increase in the explained variance. No relationship was found between the elements of the components between Spain and Colombia, and a theoretical relationship could not be established (see Appendix 1).

**Table 2:** Weights for each item selected in the factors from the Colombian and Spanish samples.

	**Spanish sample**				**Colombian sample**			
**Items**	**1**	**2**	**3**	**4**	**1**	**2**	**3**	**4**
28	0.771				0.719			
27	0.728				0.622			
26	0.661				0.598			
25	0.592				0.708			
29	0.588				0.66			
35	0.377				0.359			
57		0.804				0.697		
56		0.753				0.75		
49		0.576				0.509		
59		0.355				0.468		
51			0.816				0.690	
36			0.749				0.692	
50			0.551				0.57	
52			0.524				0.637	
40			0.481				0.534	
53				0.807				0.800
54				0.753				0.809
55				0.748				0.755
9				0.346				0.534

After analyzing the factors, the dimensions were refined as follows: (1) self-organization; (2) critical thinking; (3) understanding context, and (4) socio-political engagement. The distribution of the items that comprise the four dimensions, in the procedural and contextual frameworks that form part of the theoretical proposal of this study (see Study 1) can be observed in Table 3.

**Table 3:** Distribution of the definitive items for the proposed dimensions and theoretical frameworks.

**Dimension**	**Procedural framework**			**Contextual framework**		
	**Self-reflection**	**Decision making**	**Assuming consequences**	**Intra**	**Inter**	**Trans**
Self- organization		26		26		
	27			27		
	28			28		
	29			29		
			35	35		
			25	45		
Critical thinking		49				49
	27		56		56	
		57				57
			59		59	
Understanding context			50			50
	51					51
		36				36
		52				52
	40					40
Socio-political engagement	9					9
		53				53
			54			54
		55				55

The exploratory analyses were repeated, but this time only with the items of the first four factors identified. As a result, it was observed that the amount of variance explained by the factors increased to 53%. Cronbach's alpha for this final set of items was 0.84 for the total sample (0.83 for Spain and 0.84 for Colombia) and for each of the factors identified was 0.80 (self-organization), 0.70 (critical thinking), 0.74 (understanding context), and 0.77 (socio-political engagement).

#### Rasch Model Analysis

In the Rasch model it was observed that the item separation index was quite similar in both groups: 10.05 for the Colombian sample and 10.04 for the Spanish sample, indicating that the items discern between different levels of autonomy for the subjects in the study.

Global reliability or Item Separation Reliability was excellent (0.99). The separation index for the subjects is 2.21 for the Colombian sample and 2.24 for the Spanish sample, which indicates the extent to which the test is able to discern differences in the sample at sufficient levels for the purpose of the study. In this case, the values obtained indicate that in the samples studied, EDATVA is able to discern at least two levels (subjects with low and high autonomy). The average person reliability index (Person Separation Reliability) for both samples was appropriate (0.83 in both cases).

Consequently, in Study 2 the number of items in the EDATVA scale was significantly reduced, from 61 to 19, and the structure modified, from three to four dimensions. The scale proved to be a good model, which adapted to the Spanish-Colombian sample. Specifically: (1) the exploratory factorial analysis gave rise to four consistent factors, which form part of the theoretical proposal of the study; (2) the Rasch model verified that the items discern differences at least between two levels of autonomy, subjects with low and high autonomy, and (3) Cronbach's alpha for the final set of 19 items was 0.84 for the total sample.

Lastly, it should be noted that the EDATVA items were generated after an exhaustive validation process to adapt them to the construct and the population under study. The measure was specifically adapted to the linguistic and cultural characteristics of the Colombian and Spanish populations, which may favor the application and comparison of the results in different territories—especially the Spanish—speaking population—always taking into account social and cultural characteristics (see Appendix 2).

## Conclusion and General Discussion

In conclusion, the 19-item EDATVA is a reliable and valid model for measuring the autonomy of young people in their transition to adulthood, between the ages of 16 and 21. It presents a structure of four dimensions, which are vital factors for attaining autonomy in the transition to adulthood: (1) self- organization; (2) understanding context; (3) critical thinking, and (4) socio-political engagement. These dimensions are expressed as capacities for autonomy, which we understand in the sense proposed by Oshana (2016), who argues that autonomy “consists of the minimum qualities a person must possess in order to lead a self-governed life” (p. 6). Suárez et al. (2007) complete this concept of capacities highlighting that attaining autonomy involves “complex psychological formations […] with a predominantly executor character, the premise and result of the successful realization of a subject's action and the creation of something new” (p. 32–33).

The model has a clear and well-defined structure, which was verified during the study, as well as excellent reliability (internal consistency), a satisfactory item separation index, and test-item correlations within the recommended values. Similarly, the indices calculated from the Rasch model were also adequate. This was observed in both the Spanish and Colombian samples.

EDATVA was designed as a comprehensive and multidimensional scale, from an approach that balances intra, inter and trans-subjective elements based on the knowledge acquired in a systematic, international and interdisciplinary process of reviewing the literature and measures on autonomy.

In reference to the dimensions recognized in EDATVA, the first two—self-organization and critical thinking—comprise items present in most of the autonomy models reviewed: self-organization is one of the main aspects contemplated in the characterization and measurement of autonomy, while critical thinking is also contemplated from different perspectives. However, the latter dimension provides a novel perspective, with a clear inter and trans-subjective component, which contemplates the rights of the subject and social justice as references. This is represented by the items that deal with the defense of one's own rights in decision making or the response to their violation.

In turn, the dimensions of understanding context and socio-political engagement incorporate a series of elements that not only refer to autonomy as a product of interactions with others, but also take into consideration interactions with the environment and the limits generated on a subject's autonomy by the different contexts in which they interact: the system in which they live. In this regard, this is the first time that autonomy has been measured on a scale that clearly and predominantly incorporates approaches and elements such as these, fundamentally trans-subjective. This is in line with proposals by authors already mentioned in the introduction to this study, and others such as Thomas and Da Costa (2014), who identify autonomy as cooperation with others; D'Angelo (2013), who argues that autonomy implies “interlinking” with contexts, and Cáliz et al. (2013), who consider that decision making implies reaching a balance between one's own well-being and that of others. In this regard, autonomy is not an individual characteristic, but emerges from interdependent relationships (Parron, 2014). As Morin states: “the notion of human autonomy is complex because it depends on cultural and social conditions […] as such we make our own choices within the assortment of existing ideas and reflect independently…” (Morin, 2005, p. 97–98).

Some of the measures of autonomy reviewed also present similar elements, although in a more limited way. Worth mentioning is the study by Moleiro et al. (2016); the Autonomy- Connectedness Scale (ACS-30) by Bekker and van Assen (2006), which includes a subscale on “sensitivity to others,” and the EDPS/74 questionnaire which analyzes the subject's participation in social life. Although none of them incorporates the trans-subjective perspective in the same in-depth way as EDATVA, which incorporates items referring to events “in my country” as important data for making future decisions, or the idea that the community in which the subject lives “improves if I'm part of the activities that happen there” or if “I've got initiative to carry out proposals or ideas” that can improve it.

EDATVA therefore aims to contribute significantly to the knowledge and understanding of autonomy and, in this regard, provides a valuable model for research based on a construct with optimal psychometric properties that provide an alternative way of understanding autonomy. It also promotes the gathering of objective and consistent data on the characteristics of young people with different profiles and from different environments in their transition to adulthood.

Lastly, due to its characteristics this measure can be used in the diagnosis, orientation, and assessment of individuals, families and groups in the fields of psychology, education, sociology and/or social work.

In the case of the relationship between autonomy and the family, numerous studies highlight the influence of family educational styles and certain family characteristics on young people's autonomy (Ponciano and Féres-Carneiro, 2014; Campione-Barr et al., 2015; Esteinou, 2015; Van Petegem et al., 2015). With reference to the relationship between autonomy and formal education, research has highlighted its influence on academic success and on the role of teachers (De Carvalho and De Almeida, 2011; Bustamante et al., 2014; Hein and Jõesaar, 2015).

EDATVA can also support the design of strategies in the field of psycho-social-educational intervention with young people. In this regard, it is important to consider that this model is targeted primarily at the younger population (16–21), although it could be very useful for tackling different types of actions with young people at risk. Research can also be found on autonomy relating to socio- educational intervention with this study population, as well as social protection programs and the acquisition of personal, social and functional skills (Barker, 2014; Gibson and Cartwright, 2014; Livindo De Senna Corrêa et al., 2014; Antunes and Correia, 2016; Longas and Riera, 2016; Marques et al., 2016; Zamith-Cruz et al., 2016; Pini and Valore, 2017).

Although EDATVA has many strengths, it also has some limitations. Evidently, by selecting the initial samples for our target population the results cannot be generalized to populations in different cultures, times or age groups. Future studies replicating EDATVA will therefore be required to continue exploring its reliability and validity in larger samples and with different sociocultural groups. In turn, and unlike most other measures of autonomy, EDATVA contemplates intrasubjective aspects in a more limited way. This was a conscience decision made by the research team, nonetheless new versions of the scale could contribute important aspects to the field.

Lastly, it should be noted that the language used in the 19-item EDATVA is that same language used by young people themselves, which has proved to be easy for them to understand. The scale is easy to use and interpret, especially considering the age range of the target population and its possible uses within the contexts of assessing and intervening in young people's behavior.

## Data Availability Statement

The data supporting the conclusions of this paper will be made available to any professional researcher without undue reservation.

## Ethics Statement

The studies involving human participants were reviewed and approved by the Ethics Committee of the Universidad Santo Tomás (Bogotá, Colombia) and the Ethics Committee of the Universidad Nacional de Educación a Distancia (Madrid, Spain). Written informed consent to participate in this study was provided by the participants, or their legal guardian/next of kin in the case of minors.

## Author Contributions

TB led the project and initiated the model design, with the support of MM, and with the collaboration of the research team. CC created the database and performed the statistical analysis. TB and CC wrote the first draft of the article, which was later reviewed by all three authors. TB and MM obtained financial support for the study.

### Conflict of Interest

The authors declare that the research was conducted in the absence of any commercial or financial relationships that could be construed as a potential conflict of interest.
