# Supplementary material for: Transition to Adulthood Autonomy Scale for Young People: Design and Validation
Source: Front Psychol. 2020 Mar 20;11:457. doi: 10.3389/fpsyg.2020.00457 (PMC7100080; doi:10.3389/fpsyg.2020.00457)
Supplement: Supplementary file 1 [file Table_1.pdf]

APPENDIX 1 - ROTATED COMPONENT MATRIX (SPAIN AND COLOMBIA)

Spain

	Component																
	1	2	3	4	5	6	7	8	9	10	11	12	13	14	15	16	17
EA28	,771																
EA27	,728																
EA26	,661																
EA25	,592						,302										
EA29	,588																
EA2	,424																
EA5	,363										,312						
EA57		,804															
EA56		,753															
EA49		,576															
EA59		,355											,351				
EA51			,816														
EA36			,749														
EA50			,551														
EA52			,524														
EA40			,481														
EA53				,807													
EA54				,753													
EA55				,748													
EA11					,718												
EA14					,621												
EA13					-,602												
EA10					,565												
EA12					,466			,345		,301							
EA19						,727											
EA17						,678											
EA60						,655											
EA18						-,611											
EA7							,708										
EA6							,613										
EA8							,553										
EA9				,346			,432										
EA41								,755									
EA38								,511							,393		

EA58								,444									
EA46								,380									
EA43									,741								
EA48									,640								
EA1										,668							
EA15										-,407							
EA20										,378						-,354	
EA4									,309	,352							
EA34											,705						
EA44											,698						
EA35	,377										,382						,331
EA33												,787					
EA22						,310						,598					
EA61													,655				
EA30													,571				
EA23													,318				
EA16														,767			
EA24														,620			
EA37															,787		
EA45															,425		
EA31																,657	
EA32																	,662

Extraction method: Principal Component Analysis.

Rotation method: Varimax standardization with Kaiser.

Colombia

	Component														
	1	2	3	4	5	6	7	8	9	10	11	12	13	14	15
EA28	,719														
EA25	,708														
EA29	,660														
EA27	,622														
EA26	,598														
EA54		,809													
EA53		,800													
EA55		,755													
EA9		,534													
EA8	,304	,514													
EA7		,462					-,391			,306					
EA36			,692												

EA51			,690												
EA52			,637												
EA50			,570	,327											
EA40			,534									,346			
EA56				,750											
EA57				,697											
EA49	,314			,509											
EA59				,468							,336				
EA58					,673										
EA45					,575										
EA46					,572										
EA41					,454										
EA38			,369		,381									,304	
EA19						,739									
EA18						-,662									
EA60					,428	,521									
EA17						,517									
EA10							,616								
EA12							,606								
EA11							,513								
EA1								,680							
EA34								,522							
EA2								,472		,319					
EA4	,326							,429							
EA35	,359							,419							
EA43									,672						
EA48									,638						
EA44									,556						
EA6										,648					
EA5										,640					
EA15						,323				,362					
EA23											,647				
EA20				,305							,455				
EA30	,341										,375				
EA32											,319				
EA16												,672			
EA24	,453											,570			
EA22													,707		
EA33													,688		
EA37														,678	

EA31								,324						,352	
EA14															,606
EA13											,323				-,595

Extraction method: Principal Component Analysis.

Rotation method: Varimax standardization with Kaiser.
