# Supplementary material for: Transition to Adulthood Autonomy Scale for Young People: Design and Validation
Source: Front Psychol. 2020 Mar 20;11:457. doi: 10.3389/fpsyg.2020.00457 (PMC7100080; doi:10.3389/fpsyg.2020.00457)
Supplement: Supplementary file 2 [file Table_2.pdf]

APPENDIX 2 - Escala de Autonomía para el Tránsito a la Vida Adulta (EDATVA) (Transition to Adulthood Autonomy Scale)

Numeración	ÌTEMS
9	Mi comunidad mejora si formo parte de las actividades que se hacen en ella
25	Cuando estoy realizando una tarea nueva para mí, pienso en lo que puedo aprender de ella
26	Me gusta aprender cosas nuevas, aunque me resulte difícil
27	Me organizo de forma eficaz para desarrollar una actividad
28	Me organizo bien para aprender cosas nuevas
29	Reflexiono sobre lo que estoy aprendiendo
35	Me esfuerzo para hacer realidad mis planes de futuro
36	Lo que ocurre en mi país es importante para que yo tome decisiones sobre mi futuro
40	Estudio las propuestas de los candidatos cuando voy a participar en una votación
49	Defiendo mis derechos cuando tomo decisiones importantes
50	Es importante mostrar una actitud firme ante las decisiones políticas que me afecten
51	Pensar en los problemas que afectan a mi país es importante para mi futuro
52	Para tomar una postura sobre un problema del país, busco información en diferentes medios
53	Participo en la organización de actividades en mi comunidad
54	Formo parte de asociaciones u organizaciones sociales porque benefician a todos
55	Tengo iniciativa para llevar a cabo propuestas o ideas que puedan mejorar las condiciones de mi comunidad
56	Cuando vulneran mis derechos hago algo para recuperarlos
57	Utilizo los mecanismos disponibles para reclamar lo que me parece injusto

Numeración	ÌTEMS
59	Es importante manifestar las ideas que tienes, aunque la pareja se pueda molestar
